# Supplementary material for: Higher-order and fractional discrete time crystals in clean long-range interacting systems
Source: Nat Commun. 2021 Apr 20;12:2341. doi: 10.1038/s41467-021-22583-5 (PMC8058086; doi:10.1038/s41467-021-22583-5)
Supplement: Supplementary file 1 — Supplementary Information [file 41467_2021_22583_MOESM1_ESM.pdf]

Supplementary Information for “Higher-order and fractional discrete time crystals in clean long-range interacting systems”

Andrea Pizzi[1], Johannes Knolle[2,3,4] and Andreas Nunnenkamp[5]
[1] *Cavendish Laboratory, University of Cambridge, Cambridge CB3 0HE, United Kingdom*
[2] *Department of Physics, Technische Universität München, James-Franck-Straße 1, D-85748 Garching, Germany*
[3] *Munich Center for Quantum Science and Technology (MCQST), 80799 Munich, Germany*
[4] *Blackett Laboratory, Imperial College London, London SW7 2AZ, United Kingdom and*
[5] *School of Physics and Astronomy and Centre for the Mathematics and Theoretical Physics of Quantum Non-Equilibrium Systems, University of Nottingham, Nottingham, NG7 2RD, United Kingdom*

The Supplementary Information are devoted to technical details of the derivations and complimentary results and is organized as follows. In Supplementary Note 1 we show the mapping of the Lipkin-Meshkov-Glick (LMG) model for fully-connected spins to a model of bosons in a double well, and exploit it to obtain the Gross-Pitaevskii equation (GPE) in the limit of inifinite number of spins N. In Supplementary Note 2 we present results from exact diagonalization, showing how the discrete time crystals (DTCs) emerge for an increasing number of spins N. We show that higher-order n-DTCs emerge at much larger N as compared to the standard 2-DTC, which explains the difficulty to numerically observe them. In Supplementary Note 3 we give an overview of the spin-wave approximation we employ, whereas in Supplementary Note 4 we show that a binary Floquet protocol also leads to results similar to the ones of the continuous driving discussed in the main text. In Supplementary Note 5 we present an analysis on the stability of the higher-order n-DTCs in the presence of a longitudinal field breaking the $\mathbb{Z}_2$ symmetry of the Hamiltonian, showing that the absolute stability persists in the LMG limit and the expected prethermal nature of the DTC beyond this limit is only changed quantitatively.

Supplementary Note 1: GROSS-PITAEVSKII EQUATION

In this Note, we derive the semiclassical GPE of motion for the LMG model of N fully-connected spins ($\lambda = \alpha = 0$). To this end, we first map the spin system into a model of bosons in a double well. We then derive Heisenberg equations for the bosonic operators, and we treat them semiclassically replacing the bosonic operators with complex numbers.

Map to bosons in a double well

The Schwinger's oscillator model of angular momentum connects the algebra of angular momentum and the algebra of two bosonic modes [?]. Here, we show this connection explicitly for the collective spin of a system of N spin-1/2.

Given $p = (p_1, p_2, \ldots, p_N)$ a permutation of the indexes $1, 2, \ldots, N$, we say P_p the permutation operator acting as

$$P_p |s_1, s_2, \ldots, s_N\rangle = |s_{p_1}, s_{p_2}, \ldots s_{p_N}\rangle, \tag{1}$$

where $s_i \in \{\uparrow, \downarrow\}$. The permutation operators are used to build the symmetrization operator $\mathcal{S}$, defined as

$$\mathcal{S} = \frac{1}{\sqrt{N!}} \sum_p P_p. \tag{2}$$

We say $|n_\uparrow, n_\downarrow\rangle$ the symmetrized state with $n_\uparrow$ spins up and $n_\downarrow$ spins down, that is

$$|n_\uparrow, n_\downarrow\rangle = \frac{1}{\sqrt{n_\uparrow! n_\downarrow!}} \mathcal{S} |\underbrace{\uparrow, \uparrow, \ldots, \uparrow}_{n_\uparrow}, \underbrace{\downarrow, \downarrow, \ldots, \downarrow}_{n_\downarrow}\rangle. \tag{3}$$

Since N is fixed and $n_\downarrow = N - n_\uparrow$, the notation $|n_\uparrow, n_\downarrow\rangle$ is actually slightly redundant. In the following, we nevertheless prefer to stick with this notation for the sake of clarity. The states $|n_\uparrow, n_\downarrow\rangle$ form a basis for the Hilbert subspace of completely symmetrized states. Given an operator O commuting with the symmetrization operator $\mathcal{S}$, the action of O on this subspace is therefore fully-characterized by its action on the states $|n_\uparrow, n_\downarrow\rangle$.

In particular, we consider the 'collective' operators $\left(\sum_{j=1}^N \sigma_j^\alpha\right)$ with $\alpha = x, y, z$, which indeed all commute with $\mathcal{S}$. We have

$$\begin{aligned}
\left(\sum_{j=1}^N \sigma_j^x\right) |n_\uparrow, n_\downarrow\rangle =& \frac{1}{\sqrt{n_\uparrow! n_\downarrow!}} \mathcal{S} \sum_{j=1}^N \sigma_j^x |\underbrace{\uparrow, \ldots, \uparrow}_{n_\uparrow}, \underbrace{\downarrow, \ldots, \downarrow}_{n_\downarrow}\rangle & (4)\\
=& \frac{1}{\sqrt{n_\uparrow! n_\downarrow!}} \sum_{j=1}^{n_\uparrow} \mathcal{S} |\underbrace{\uparrow, \ldots, \uparrow}_{n_\uparrow - 1}, \underbrace{\downarrow, \ldots, \downarrow}_{n_\downarrow + 1}\rangle + \frac{1}{\sqrt{n_\uparrow! n_\downarrow!}} \sum_{j=n_\uparrow+1}^{N} \mathcal{S} |\underbrace{\uparrow, \ldots, \uparrow}_{n_\uparrow + 1}, \underbrace{\downarrow, \ldots, \downarrow}_{n_\downarrow - 1}\rangle & (5)\\
=& n_\uparrow \frac{\sqrt{(n_\uparrow - 1)!(n_\downarrow + 1)!}}{\sqrt{n_\uparrow! n_\downarrow!}} |n_\uparrow - 1, n_\downarrow + 1\rangle + n_\downarrow \frac{\sqrt{(n_\uparrow + 1)!(n_\downarrow - 1)!}}{\sqrt{n_\uparrow! n_\downarrow!}} |n_\uparrow + 1, n_\downarrow - 1\rangle & (6)\\
=& \sqrt{n_\uparrow(n_\downarrow + 1)}\, |n_\uparrow - 1, n_\downarrow + 1\rangle + \sqrt{(n_\uparrow + 1) n_\downarrow}\, |n_\uparrow + 1, n_\downarrow - 1\rangle\,, & (7)
\end{aligned}$$

and, similarly,

$$\begin{aligned}
\left(\sum_{j=1}^N \sigma_j^y\right) |n_\uparrow, n_\downarrow\rangle =& \frac{i}{\sqrt{n_\uparrow! n_\downarrow!}} \sum_{j=1}^{n_\uparrow} \mathcal{S} |\underbrace{\uparrow, \ldots, \uparrow}_{n_\uparrow - 1}, \underbrace{\downarrow, \ldots, \downarrow}_{n_\downarrow + 1}\rangle - \frac{i}{\sqrt{n_\uparrow! n_\downarrow!}} \sum_{j=n_\uparrow+1}^{N} \mathcal{S} |\underbrace{\uparrow, \ldots, \uparrow}_{n_\uparrow + 1}, \underbrace{\downarrow, \ldots, \downarrow}_{n_\downarrow - 1}\rangle & (8)\\
=& i\sqrt{n_\uparrow(n_\downarrow + 1)}\, |n_\uparrow - 1, n_\downarrow + 1\rangle - i\sqrt{(n_\uparrow + 1) n_\downarrow}\, |n_\uparrow + 1, n_\downarrow - 1\rangle & (9)
\end{aligned}$$

and, finally,

$$\left(\sum_{j=1}^N \sigma_j^z\right) |n_\uparrow, n_\downarrow\rangle = (n_\uparrow - n_\downarrow)\, |n_\uparrow, n_\downarrow\rangle\,. \tag{10}$$

Introducing standard bosonic operators $a_\uparrow, a_\downarrow, a_\uparrow^\dagger, a_\downarrow^\dagger$ for the two bosonic modes labeled by $\uparrow$ and $\downarrow$, we can thus write

$$\sum_{j=1}^{N} \sigma_j^x = a_\uparrow^\dagger a_\downarrow + a_\downarrow^\dagger a_\uparrow, \qquad \sum_{j=1}^{N} \sigma_j^y = -i\left(a_\uparrow^\dagger a_\downarrow - a_\downarrow^\dagger a_\uparrow\right), \qquad \sum_{j=1}^{N} \sigma_j^z = n_\uparrow - n_\downarrow \tag{11}$$

with $n_\uparrow = a_\uparrow^\dagger a_\uparrow$ and $n_\downarrow = a_\downarrow^\dagger a_\downarrow$.

The Hamiltonian (1) in the LMG limit ($\alpha = \lambda = 0$) reads

$$H = \frac{J}{N}\sum_{i,j}^{N} \sigma_i^z \sigma_j^z - \pi h[1+\sin(2\pi t)]\sum_{j=1}^{N} \sigma_j^x, \tag{12}$$

and is thus rewritten in terms of the bosonic operators as

$$H = \frac{J}{N}\left(n_\uparrow - n_\downarrow\right)^2 - \pi h[1+\sin(2\pi t)](a_\uparrow^\dagger a_\downarrow + a_\downarrow^\dagger a_\uparrow). \tag{13}$$

We elaborate on the first term of Supplementary Eq. (13) using $m = \frac{n_\uparrow - n_\downarrow}{N}$ and noting that

$$\begin{cases} \frac{n_\uparrow}{N} = \frac{1+m}{2} \\ \frac{n_\downarrow}{N} = \frac{1-m}{2} \end{cases} \qquad \Rightarrow \qquad \frac{n_\uparrow n_\downarrow}{N^2} = \frac{1-m^2}{4}, \tag{14}$$

from which

$$m^2 = \left(\frac{n_\uparrow - n_\downarrow}{N}\right)^2 = \frac{n_\uparrow^2 + n_\downarrow^2 - 2n_\uparrow n_\downarrow}{N^2} = \frac{n_\uparrow^2 + n_\downarrow^2}{N^2} + \frac{m^2}{2} - \frac{1}{2}, \tag{15}$$

and, isolating m^2,

$$m^2 = 2\frac{n_\uparrow^2 + n_\downarrow^2}{N^2} - 1 = 2\frac{n_\uparrow(n_\uparrow - 1) + n_\downarrow(n_\downarrow - 1)}{N^2} - 1 + \frac{2}{N}. \tag{16}$$

Setting $U = 4J$ and $\tau(t) = \pi h[1+\sin 2\pi t]$, up to irrelevant additional constant terms, the Hamiltonian thus reads

$$H(t) = -\tau(t)(a_\uparrow^\dagger a_\downarrow + a_\downarrow^\dagger a_\uparrow) + \frac{U}{2N}\big(n_\uparrow(n_\uparrow - 1) + n_\downarrow(n_\downarrow - 1)\big), \tag{17}$$

where $n_\uparrow = a_\uparrow^\dagger a_\uparrow$ and $n_\downarrow = a_\downarrow^\dagger a_\downarrow$. That is, the LMG model in the symmetric subspace is mapped to a model for N bosons in a double well (the two wells being labeled by $\uparrow$ and $\downarrow$).

Equations of motion

The bosonic representation of Supplementary Eq. (17) is particularly convenient to obtain dynamical equations. The Heisenberg equations for the bosonic operators read ($\hbar = 1$)

$$\begin{aligned} \frac{da_\uparrow}{d(it)} &= [H(t), a_\uparrow] = \tau(t)a_\downarrow - \frac{U}{N}n_\uparrow a_\uparrow, \\ \frac{da_\downarrow}{d(it)} &= [H(t), a_\downarrow] = \tau(t)a_\uparrow - \frac{U}{N}n_\downarrow a_\downarrow. \end{aligned} \tag{18}$$

In the limit $N \to \infty$, upon replacing $a_\uparrow \to \sqrt{N}\psi_\uparrow$ and $a_\downarrow \to \sqrt{N}\psi_\downarrow$, with $\psi_\downarrow$ and $\psi_\downarrow$ complex fields, we finally derive the following Gross-Pitaevskii equation

$$\begin{aligned} \frac{d\psi_\uparrow}{d(it)} &= \tau(t)\psi_\downarrow - U|\psi_\uparrow|^2\psi_\uparrow, \\ \frac{d\psi_\downarrow}{d(it)} &= \tau(t)\psi_\uparrow - U|\psi_\downarrow|^2\psi_\downarrow. \end{aligned} \tag{19}$$

For an operator $\hat{O} = f(a_\uparrow, a_\downarrow, a_\uparrow^\dagger, a_\downarrow^\dagger)$ written as a function f of the bosonic operators, the beyond-mean-field dynamics of the expectation value $O(t) = \langle \hat{O} \rangle(t)$ can be generally computed within a Truncated Wigner approximation (TWA) as

$$O(t) \approx \langle f(\psi_\uparrow(t), \psi_\downarrow(t), \psi_\uparrow^*(t), \psi_\downarrow^*(t)) \rangle_{\psi_\uparrow(0),\psi_\downarrow(0)} \tag{20}$$

where $\langle \dots \rangle_{\psi_\downarrow(0),\psi_\uparrow(0)}$ denotes the average over an ensemble of stochastic semiclassical initial conditions $\psi_\uparrow(0)$ and $\psi_\downarrow(0)$ that are drawn according to the quantum initial condition, and then evolve in time with the GPE (19).

In particular, let us consider as initial condition the symmetrized state $|\psi'(0)\rangle$ with magnetization $m' = 1 - \delta$, with $0 < \delta \ll 1$ and $m'N$ integer (which, since we assume $N \to \infty$, does not restrict the possible values for m')

$$|\psi'(0)\rangle = |n'_\uparrow, n'_\downarrow\rangle, \qquad n'_\uparrow = m'N, \; n'_\downarrow = (1 - m')N, \tag{21}$$

for which the TWA is performed considering the following ensemble of initial conditions

$$\psi'_\uparrow(0) = \sqrt{1 - \frac{\delta}{2}} e^{i\theta_\uparrow(0)}, \qquad \psi'_\downarrow(0) = \sqrt{\frac{\delta}{2}} e^{i\theta_\downarrow(0)}, \tag{22}$$

with $\theta_\uparrow(0)$ and $\theta_\downarrow(0)$ independent uniform random numbers between 0 and 2π. Thanks to a gauge transformation, we can always change the initial conditions (22) into

$$\psi'_\uparrow(0) = \sqrt{1 - \frac{\delta}{2}}, \qquad \psi'_\downarrow(0) = \sqrt{\frac{\delta}{2}} e^{i\theta_0}, \tag{23}$$

where θ_0 is also a random number between 0 and 2π. Consider now the limit of $\delta \to 0$, that is of $|\psi'(0)\rangle \to |\psi(0)\rangle = |\uparrow, \uparrow, \dots, \uparrow\rangle$. In this limit, the ensemble of stochastic initial conditions (23) shrinks in the phase space of complex coordinates $\psi'_\uparrow(0)$ and $\psi'_\downarrow(0)$ towards the point $\psi_\uparrow(0) = 1 - \psi_\downarrow(0) = 1$. If the GPE is nonchaotic, the points of the shrinked ensemble follow close trajectories, so that the TWA average in Supplementary Eq. (20) can be actually replaced by the evaluation of f for a single trajectory of the ensemble, say the one starting in $\psi_\uparrow(0) = 1 - \psi_\downarrow(0) = 1$. In contrast, if the GPE is chaotic, because of sensitivity to initial conditions, the ensemble quickly spreads, scrambling across the classical phase space. In this case the ensemble trajectories at long-times in (20) interfere destructively, washing out any time oscillation of O: the TWA results in thermalization.

In the limit $|\psi'(0)\rangle \to |\psi(0)\rangle = |\uparrow, \uparrow, \dots, \uparrow\rangle$, it is therefore convenient to consider the following 'single-shot GPE' [?], to be run just once

$$O(t) \to \big(f(\psi_\uparrow(t), \psi_\downarrow(t), \psi_\uparrow^*(t), \psi_\downarrow^*(t))\big)_{\psi_\uparrow(0)=1,\psi_\downarrow(0)=0}, \tag{24}$$

which is then expected to be accurate when nonchaotic, and to signal quantum thermalization when chaotic, which motivates the use of the symbol " $\to$ ", rather than " $=$ ".

In particular, considering the observable $\vec{S} = \frac{1}{N}\sum_{j=1}^N \vec{\sigma}_j$, from Supplementary Eq. (11) we thus write

$$\frac{\langle S^x \rangle + i\langle S^y \rangle}{2} \to \psi_\uparrow^* \psi_\downarrow, \qquad \langle S^x \rangle \to |\psi_\uparrow|^2 - |\psi_\downarrow|^2. \tag{25}$$

Supplementary Note 2: EXACT DIAGONALIZATION

The LMG model is also particularly suitable for exact diagonalization techniques. In fact, the size of the symmetric subspace $(N+1)$ scales only linearly with the system size N, making exact diagonalization viable for systems which are much larger than the ones typically considered in less-symmetric 1D models. In Fig. 1 we show the Fourier transform $\tilde{m}(\nu)$ of the magnetization $m = \langle \sigma_j^z \rangle$ for an initially $\mathbf{z}$-polarized state $|\psi(0)\rangle = |N_\uparrow\rangle = |\uparrow, \uparrow, \dots, \uparrow\rangle$. For increasing N, we can observe the emergence of the constant-frequency plateaus signaling the n-DTCs. First, this confirms that the GPE captures the correct dynamics for large N, as expected. Second, we notice that, for the 2-DTC and the 4-DTC, the plateau is clearly visible only for $N \gtrapprox 10$ and $N \gtrapprox 100$, respectively. This is remarkable, as it suggests that higher-order DTCs may be numerically harder to observe as they could appear at larger system sizes, generally beyond the reach of exact diagonalization techniques. This observation might explain why higher-order DTCs have so far remained elusive to numerical examples [?].

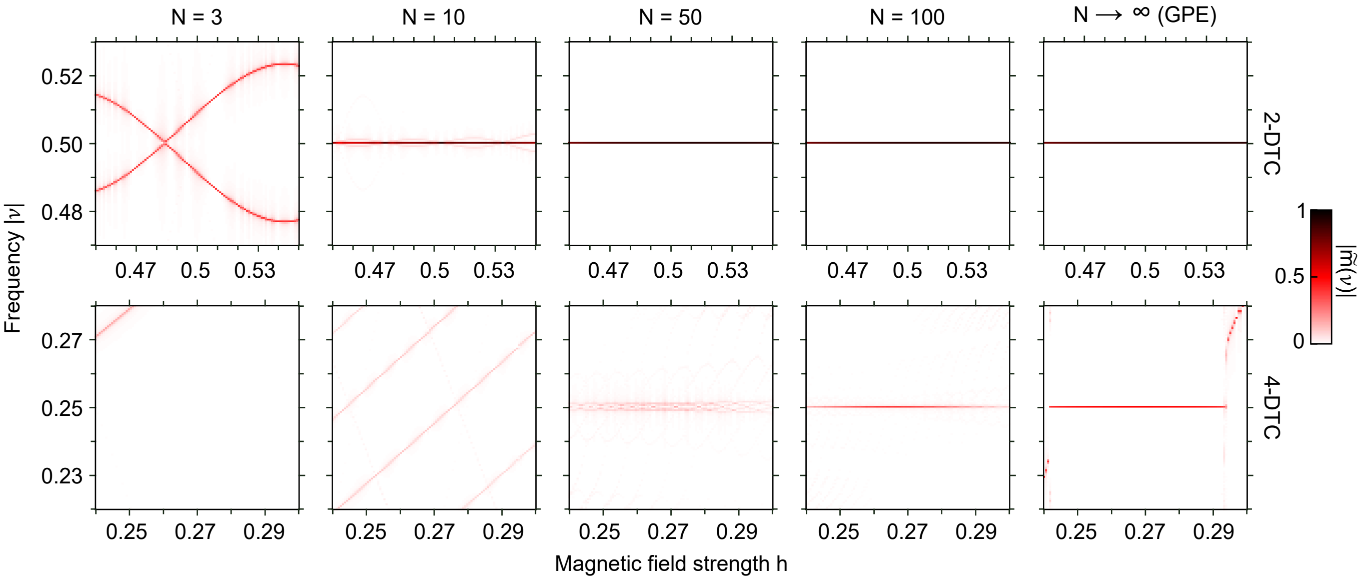

Supplementary Fig. 1: **Exact diagonalization and system size scaling**. For an initially **z**-polarized state $|\psi(0)\rangle = |\uparrow, \uparrow, \ldots, \uparrow\rangle$ we plot the Fourier transform $\tilde{m}(\nu)$ of the magnetization $m = \langle \sigma_j^z \rangle$ for various number of spins N and a fixed interaction strength $J = 0.5$. Results from the GPE in the limit $N \to \infty$ are reported on the right as a reference. (top row) For $h \approx 0.5$, the 2-DTC plateau at frequency 0.5 emerges for increasing N, being clearly visible already for $N \gtrapprox 10$. (bottom row) For $h \approx 0.25$, the 4-DTC plateau at frequency 0.25 also emerges for increasing N, but it does so only for considerably larger system sizes $N \gtrapprox 100$, which might explain the difficulties in observing higher-order DTCs.

Supplementary Note 3: SPIN-WAVE APPROXIMATION

In this Note, we briefly summarize the idea behind the spin-wave approximation, which is thoroughly explained in Refs. [**?** **?**] and the supplementary material therein, to which we redirect the reader for further details.

In a DTC evolving from an initially **z**-polarized state $|\psi(0)\rangle = |\uparrow, \uparrow, \ldots, \uparrow\rangle$, the spins are mostly aligned at all times. Imperfections in the alignment can be described within a Holstein-Primakoff transformation in terms of bosonic spin-wave quasiparticles $b_k^\dagger$. Crucially, the collective spin $\vec{S} = \frac{1}{N}\sum_{j=1}^N \vec{\sigma}_j$ rotates as a function of time in the lab frame. Therefore, the Holstein-Primakoff transformation has to be performed in a rotating frame (X, Y, Z) such that the, say, Z axis tracks the orientation of the collective spin at all times. This tracking is encoded in the condition $\langle S^X \rangle = \langle S^Y \rangle = 0$, from which the dynamics of the rotating frame is obtained self-consistently. On top of this, an approximation is made in that the Hamiltonian is expanded to lowest non-trivial order in the density of spin-wave excitations $\epsilon = \frac{2}{N}\sum_{k\neq 0}^N \langle b_k^\dagger b_k \rangle$, which should remain $\ll 1$ for the approximation to be consistent. This procedure results in a set of $2N$ ordinary differential equations similar to Eqs. (26) and (29) in the Supplemental Material of Ref. [**?**], describing the rotation of the new reference frame and the dynamics of the spin waves at the various momenta.

Finally, we remark the main differences between the implementation of the spin-wave approximation in our work and in Ref. [**?**]. First, Ref. [**?**] considers a constant-in-time Hamiltonian, whereas the parameters of the Hamiltonian in the present work are time-dependent. As a consequence, the parameters in the system of ordinary differential equations become time-dependent. Second, Ref. [**?**] considers a nearest-neighbor interaction on top of a fully-connected one, whereas we consider the more general case of nearest-neighbor interaction on top of a power-law one. The $\cos k$ that appears in the equations of Ref. [**?**] is therefore substituted by a more generic $\tilde{\mathcal{J}}_k = \sum_{j=1}^N \mathcal{J}_{r_{1,j}} e^{-ir_{1,j}k}$ in ours, where $\mathcal{J}_{r_{i,j}}$ contains both the nearest-neighbor and the power-law interactions.

Supplementary Note 4: BINARY DRIVING

In this Note, we compliment the results from the main paper showing that the fragmentation of the phase diagram to host a multitude of higher-order DTCs also occur for a binary Floquet protocol. In particular, we consider a

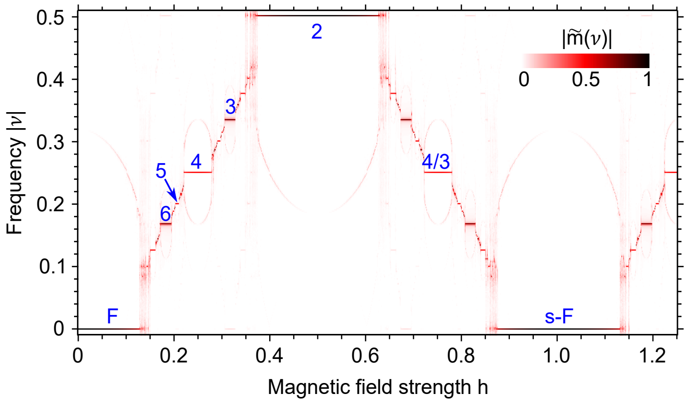

Supplementary Fig. 2: **Phase diagram fragmentation for a binary Floquet driving**. This figure is in complete analogy with Fig. 1b of the main paper, but considers the binary Floquet protocol (26). We plot the Fourier transform $\tilde{m}(\nu)$ of the magnetization $m = \langle\sigma_j^z\rangle$ in the plane of the transverse field strength h and of the frequency ν, for a fixed interaction strength $J = 0.5$ and computed over 500 periods. We observe that the spectral lines fragment in plateaus with constant frequency, signaling the n-DTCs, (dynamic) ferromagnet and stroboscopic-ferromagnet. In blue, we indicate the index n of some of the resolved DTCs.

periodic Hamiltonian with period 1 alternating fully-connected interaction and transverse field

$$H(t) = \begin{cases} +2\dfrac{J}{N}\displaystyle\sum_{i,j=1}^{N}\sigma_i^z\sigma_j^z & 0 < t \leq 0.5 \\ -2\pi h\displaystyle\sum_{j=1}^{N}\sigma_j^x & 0.5 < t \leq 1. \end{cases} \tag{26}$$

From the Hamiltonian (26), in the limit $N \to \infty$, we can derive a GPE in complete analogy with Supplementary Eq. (19). Solving it for the initially $\mathbf{z}$-polarized state $|\psi(0)\rangle = |\uparrow,\uparrow,\ldots,\uparrow\rangle$, we obtain the spectrum $\tilde{m}(\nu)$ of Fig. 2. Also for this model, it is possible to check within a spin-wave approximation that the dynamical phases persist when deviating from the LMG limit of fully-connected spins, as long as the interaction range is large enough, in complete analogy with the results of Fig. 4 of the main paper.

Supplementary Note 5: STABILITY AGAINST $\mathbb{Z}_2$ SYMMETRY-BREAKING TERMS

The Hamiltonian $H(t)$ of Eq. 1 in the main text is $\mathbb{Z}_2$ symmetric, since its commutator with a global spin flip $\prod_{j=1}^{N}\sigma_j^x$ vanishes. For a 2-DTC, a question raises whether the subharmonic response truly stems from the breaking of the time-translational symmetry, or it rather "piggybacks" on an underlying breaking of the $\mathbb{Z}_2$ symmetry[**? ? ?**]. This question motivates an analysis to assess whether the 2-DTC is stable in the presence of a term breaking the underlying $\mathbb{Z}_2$ symmetry explicitly, such as a longitudinal field. For higher-order and fractional n-DTCs, instead, no such an issue exist, because the Hamiltonian lacks any $\mathbb{Z}_n$ symmetry ($n > 2$), and the DTCs must be a "genuine" manifestation of time-symmetry breaking alone, breaking an *emergent* $\mathbb{Z}_n$ symmetry. Nonetheless, it is still interesting to investigate the effects that the explicit breaking of the $\mathbb{Z}_2$ symmetry has on the n-DTCs. For completeness, in this Note, we therefore perform a stability analysis upon introducing a longitudinal field.

Consider the following Hamiltonian in which, on top of the Hamiltonian $H(t)$ from Eq. (1) in the main text, we add a longitudinal field of strength πh_z (the factor π comes from the analogy with the definitions for the transverse field)

$$H'(t) = H(t) + \pi h_z \sum_{j=1}^{N}\sigma_j^z \tag{27}$$

In Fig. 3, we investigate the stability of the 4-DTC in the presence of the longitudinal field h_z breaking the $\mathbb{Z}_2$ symmetry of the Hamiltonian. In the LMG limit ($\alpha = \lambda = 0$), the introduction of a small field h_z has qualitatively no significant effect. In the Poincaré map, the 4 islands characterizing the 4-DTC are in fact just slightly deformed for a small h_z, suggesting that the subharmonic response lasts up to infinite time, similarly to $h_z = 0$. Away from

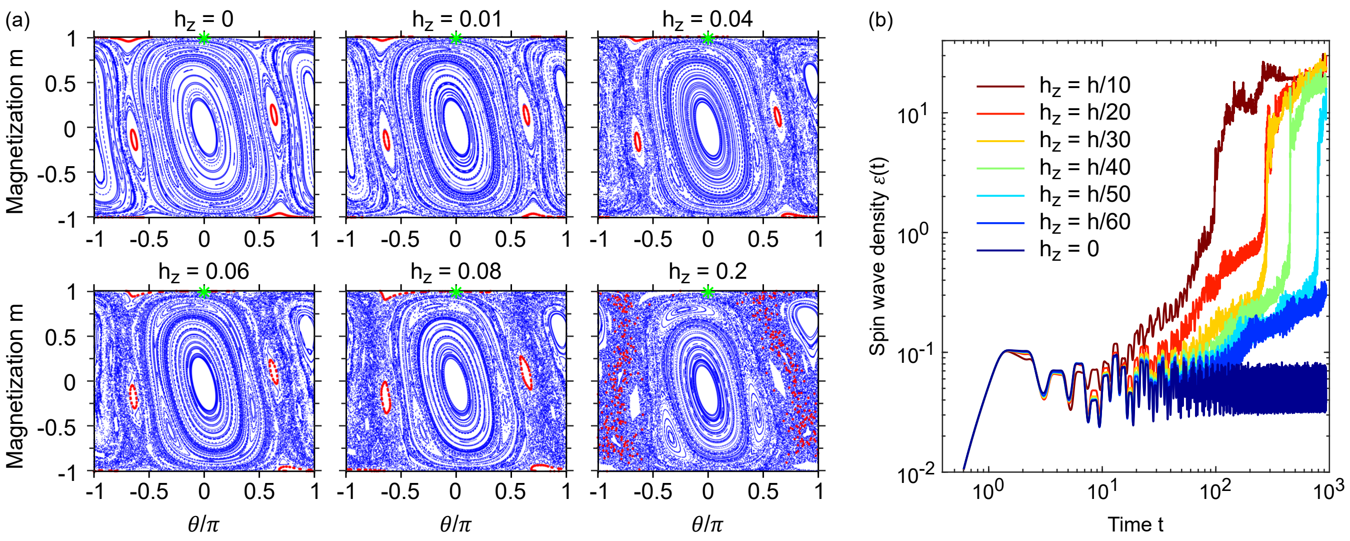

Supplementary Fig. 3: **Stability against $\mathbb{Z}_2$ symmetry-breaking terms**. We investigate the stability of the 4-DTC in the presence of a longitudinal field of strength h_z in Supplementary Eq. (27). (a) In the LMG limit, the dynamics is analysed with Poincaré maps analogue to those of Fig. 2 in our manuscript. If h_z is small enough, the islands underpinning the 4-DTC qualitatively do not change, and the 4-DTC is stable. For increasing h_z, larger and larger regions of the phase space become chaotic, and the 4-DTC is eventually broken. (b) Dynamics of the spin-wave density $\epsilon(t)$ for $\alpha = 1.2$, $\lambda = 0.03$, and $N = 1000$. The prethermal nature of the DTC is manifest in this case: spin waves proliferate after a time roughly scaling as $\sim e^{1/h_z}$. In both (a) and (b), we considered $h = 0.26$ and $J = 0.5$.

the LMG limit, instead, the dynamics within the spin-wave approximation shows a proliferation of the spin waves after a long prethermal regime. Although it is hard to assess the accuracy of this approximate method, the results suggest that the expected prethermal nature of the DTC beyond the LMG limit is only changed quantitatively, with the heating time scaling exponentially as $t_{th} \sim e^{1/h_z}$. For $h_z = 0$ we recover the results for which the 4-DTC appears infinitively long-lived within the spin-wave approximation.
